# Investigating postures and movements associated with psychological arousal and valence

**DOI:** 10.3389/fpsyg.2026.1866036

**Published:** 2026-09-14

**Authors:** Aya Takayama, Hiroshi Sekiya

**Affiliations:** 1 Faculty of Sports and Health Science, Fukuoka University, Fukuoka, Japan; 2 Graduate School of Humanities and Social Sciences, Hiroshima University, Hiroshima, Japan

**Keywords:** bodily feedback, embodiment, open-ended responses, power posing, two-dimensional model of emotion

## Abstract

**Introduction:**

Previous research has identified specific postures associated with different combinations of psychological arousal and valence. This study positioned psychological arousal and valence following different postures and movements within a two-dimensional affective space and exploratorily examined relative differences among conditions.

**Methods:**

Twenty university students (11 men and nine women) assumed six postures and performed six movements in random order for 1 min across two experimental days. Arousal and valence after each condition were assessed using the Two-Dimensional Mood Scale (TDMS). Participants also provided open-ended descriptions of the emotions experienced during each condition, and heart rate (HR) was measured as an index of physiological arousal. Linear mixed-effects models accounting for the within-participant repeated-measures structure were used for the primary analyses.

**Results:**

Differences among posture and movement conditions were observed in arousal, valence, and HR. In comparisons with the theoretical origin of the TDMS, skipping was located in the high-arousal positive quadrant, whereas the supine and meditation postures were located in the low-arousal positive quadrant. Multilevel correlation analysis showed a positive within-person association between subjective arousal and HR; however, this association was not significant in an additional linear mixed-effects model adjusting for posture and movement condition and other covariates. Open-ended responses included expressions corresponding to TDMS items as well as infrequent emotional expressions and bodily sensations not directly captured by the TDMS.

**Discussion:**

These findings provide exploratory evidence, based on a two-dimensional affective model, regarding emotional states following different postures and movements and their accompanying physiological arousal.

## 1 Introduction

Extant literature examining the effects of bodily states on psychological states has compared high-power poses (e.g., expansive postures) with low-power poses (e.g., slumped postures). In this field of research, particular attention has been paid to emotional states. Evidence indicates that compared to low-power poses, high-power poses are associated with increased positive emotions (Van Cappellen et al., 2022) and decreased negative emotions (Roberts and Arefi-Afshar, 2007). Nair et al. (2015) also found that compared to low-power poses, high-power poses increased high-arousal positive emotions and decreased low-arousal negative emotions. These findings can be explained by embodiment theory, which posits that psychological and bodily states are bidirectionally and closely interrelated ( Barsalou, 2008; Niedenthal, 2007; Winkielman et al., 2015). Embodiment theory provides a broad framework in which psychological processes, including emotion, are understood as arising from information processing within the brain as well as bodily states, sensorimotor processes, and interactions with the environment. In emotion research, this framework provides a theoretical basis for examining how bodily states, such as posture, facial expression, movement, and heart rate (HR), are associated with emotional experience. The relationship between bodily states and emotion is explained by the James–Lange theory (James, 1884; Lange, 1885/1912). According to this theory, external or internal stimuli elicit bodily responses involving the autonomic nervous system and skeletal musculature, and emotions arise through the perception of these bodily responses. Thus, a central feature of the theory is that bodily responses are positioned as a primary process preceding emotional experience. By contrast, bodily feedback theory proposes that bodily information derived from ongoing facial expressions, postures, and movements can contribute to the formation or modulation of emotional experience (Price and Harmon-Jones, 2015). The James–Lange theory seeks to explain the process through which emotions arise, whereas bodily feedback theory focuses more specifically on how adopting particular bodily states may alter emotional experience.

Although several studies have examined associations between bodily states, such as posture, and emotional states, research on power posing has primarily focused on relative comparisons between high- and low-power poses. For example, Nair et al. (2015) used the Affect Valuation Index (Tsai et al., 2006) to assess changes in four affective states—high-arousal negative, high-arousal positive, low-arousal negative, and low-arousal positive affect—and compared these changes between high- and low-power pose conditions. Thus, their analysis focused on relative differences between the two types of poses rather than on positioning each pose within a two-dimensional affective space defined by arousal and valence. By contrast, Takayama and Sekiya (2023) used the Two-Dimensional Mood Scale (TDMS; Sakairi et al., 2013) and treated the intersection of the arousal and valence axes as a theoretical neutral point to examine the position of different postures within this two-dimensional affective space. Some of the examined postures were positioned in the high-arousal negative, high-arousal positive, or low-arousal positive regions relative to the theoretical origin. However, no significant differences in TDMS scores were found between the sitting and standing postures, providing no evidence of differences between the two posture types in psychological arousal or valence.

Existing research has investigated the effects of walking on emotions. For example, skipping has been reported to increase high-arousal positive emotions more than walking in a slumped posture (Peper and Lin, 2012). Similarly, walking with an upright posture suppressed low-arousal negative emotions compared with walking with a slumped posture (Hackford et al., 2019). Furthermore, in a study by Shafir et al. (2013), participants performed movements associated with happiness, sadness, and fear, and reported their emotions before and after the movements. Results indicated that happy movements increased positive emotions, sad movements increased sadness, and fearful movements increased fear and negative emotions. Adolph et al. (2021) examined the relationship between walking patterns and emotions in everyday life, suggesting that not only posture during walking, but also factors such as walking speed and vertical up-and-down movements are associated with emotions. These findings imply that factors other than posture, such as those that occur during movement, may influence emotions. Therefore, the psychological effects of movement may extend beyond those of posture alone.

Furthermore, previous research might not have adequately assessed changes in emotions in response to posture, implying the need to reconsider how emotions are measured. For example, Takayama and Sekiya (2023) did not identify postures associated with low-arousal negative emotions. This may be because the TDMS uses terms such as “sluggish” and “lethargic” to assess this emotional category, which may not fully capture other relevant emotions. Thus, even if emotions such as “sad” or “disappointed” were experienced, they might not have been reflected in the responses. Therefore, in addition to the TDMS responses, collecting open-ended responses regarding participants' subjective experiences during posture and movement is necessary to accurately assess the emotions involved. Based on these considerations, the first aim of the present study was to explore psychological arousal and valence following different postures and movements based on a two-dimensional model of emotion. In particular, arousal and valence after each condition were positioned within the two-dimensional affective space of the TDMS, and relative differences among posture and movement conditions were examined.

In previous studies on power posing, researchers have examined the physiological effects of posture. For example, Carney et al. (2010) reported that compared to low-power poses, high-power poses increased testosterone, which is associated with confidence, and decreased cortisol—a hormone linked to stress. However, the replicability of these findings is questionable. The first study challenging the replicability of these effects (Ranehill et al., 2015) revealed that while power poses significantly influenced feelings of power, these had no significant effect on hormonal measures or behavioral outcomes such as risk-taking. Moreover, two systematic reviews using meta-analyses demonstrated consistent effects of power poses on feelings of power and other self-reported outcomes (Elkjær et al., 2022; Körner et al., 2022). However, these reviews also indicated that the hormonal effects were not reliably replicated, which was consistent with the findings of Ranehill et al. (2015). However, certain studies have focused on the autonomic nervous system activity. For example, high-power poses have been reported to increase pulse pressure, which serves as a marker of autonomic activity, compared with low-power poses under pressure (Nair et al., 2015). Additionally, from the perspective of embodiment theory, Takayama and Sekiya (2023) reported that postures involving greater physical demands, such as raising the shoulders or expanding the chest, were associated with higher HR, whereas less physically demanding postures, involving relaxed hands and arms, were associated with lower HR. Although studies grounded in embodiment theory have begun exploring the relationship between posture and autonomic nervous system activity, such studies remain limited in number and insufficient.

Furthermore, according to the James–Lange theory, emotions are believed to be influenced by physical changes due to facial expressions, posture, and movement, along with physiological changes, such as those induced by autonomic nervous system activity. For example, increased HR has been associated with higher levels of psychological arousal, whereas decreased HR has been linked to lower arousal (Kreibig, 2010; Takayama and Sekiya, 2023). However, few studies on embodiment theory have examined the influence of posture on autonomic nervous system activity, and the relationship between psychological and physiological arousal remains underexplored. Therefore, this study aims to contribute to this area by incorporating movements into its investigation. Accordingly, the second aim of this study was to compare HR across posture and movement conditions as an objective index of physiological arousal and examine its relationship with subjective arousal.

## 2 Materials and methods

### 2.1 Participants

Twenty university students (11 males, nine females; *M*_age_ = 20.15 ± 1.27) participated in the experiment. An *a priori* power analysis was conducted using G^*^Power 3.1 (Heinrich Heine University Düsseldorf, Düsseldorf, Germany)(Faul et al., 2007) to determine the required sample size for one-sample *t*-tests comparing arousal and valence scores against the theoretical origin of the TDMS, defined as the intersection of the arousal and valence axes. The mean effect size for the statistically significant results reported in Experiment 2 of Takayama and Sekiya (2023) was *d* = 0.99. However, in the present study, an expected effect size of *d* = 0.80 was specified to detect conditions showing relatively large deviations from the theoretical origin. With a two-tailed test, α = 0.05 and power (1 − β) = 0.80, the minimum required sample size was 15 participants. Therefore, we recruited 20 participants. Notably, this *a priori* power analysis was based on a single one-sample test against the theoretical origin and did not ensure sufficient statistical power for multiple-testing corrections or comparisons among posture and movement conditions. Ethical considerations were addressed by providing the participants with written documents and an oral explanation outlining the cover story, experimental procedures, handling of personal data, and their rights as participants. Based on this explanation, participants voluntarily agreed to participate. A cover story was used to conceal the true purpose of the study and minimize the demand characteristics that might have occurred if participants inferred the experimenter's intentions. Therefore, a debriefing regarding the cover story was conducted after the experiment, and the participants were compensated with 2,000 yen as a token of appreciation for their participation. None of the participants realized the true purpose of the experiment until after the debriefing. This study was approved by the Research Ethics Committee of the second author's university (Approval Number: HR-ES-000581).

### 2.2 Postures and movements

In this study, six postures and six movements were selected as candidate conditions expected to be associated with the four emotional categories in the two-dimensional model of emotion (Figure 1). Postures were selected with reference to Takayama and Sekiya (2023), who examined associations of various postures with psychological arousal and valence. Movements were selected with reference to previous studies examining associations between movements and emotions (Hackford et al., 2019; Peper and Lin, 2012; Shafir et al., 2013). In Figure 1, M denotes movements, whereas P denotes postures. Participants performed the standing movement (M5) and adopted each of the postures (P6, P8–P12) at the center of a 180 × 300 cm mat for 1 min per condition. They also performed the walking conditions (M1–M3 and M7) and the skipping condition (M4) counterclockwise around the mat for 1 min per condition. Movement tempo was standardized using a metronome application [Smart Metronome and Tuner, Version 11.0 (IHARA PRODUCTS, CO., LTD., Ichikawa, Chiba, Japan)]. The tempos were selected by considering the expected differences in arousal level and whether each movement could be performed safely and comfortably for 1 min. Based on preliminary checks conducted before the study, 140 bpm was selected for fast walking that could be performed safely; 100 bpm for walking at an approximately normal pace, backward walking, and skipping at a comfortable pace; 60 bpm for slow walking; and 40 bpm for a slow tempo at which the arm-raising movement could be repeated comfortably. Three candidate conditions were selected from the postures and movements for each emotional category. These selections were based on expected associations with the respective emotional categories; however, these expectations were not formulated as hypotheses to be tested. Instructions for all postures and movements were provided verbally, as in previous studies (Carney et al., 2010; Riskind and Gotay, 1982; Takayama and Sekiya, 2023). Between conditions, participants were instructed to adopt a seated upright posture in which the ear lobe, acromion, and greater trochanter were aligned. This posture was not intended to represent the theoretical origin of the TDMS; instead, it was used to reduce potential order and carryover effects associated with sequentially performing multiple posture and movement conditions.

**Figure 1:**
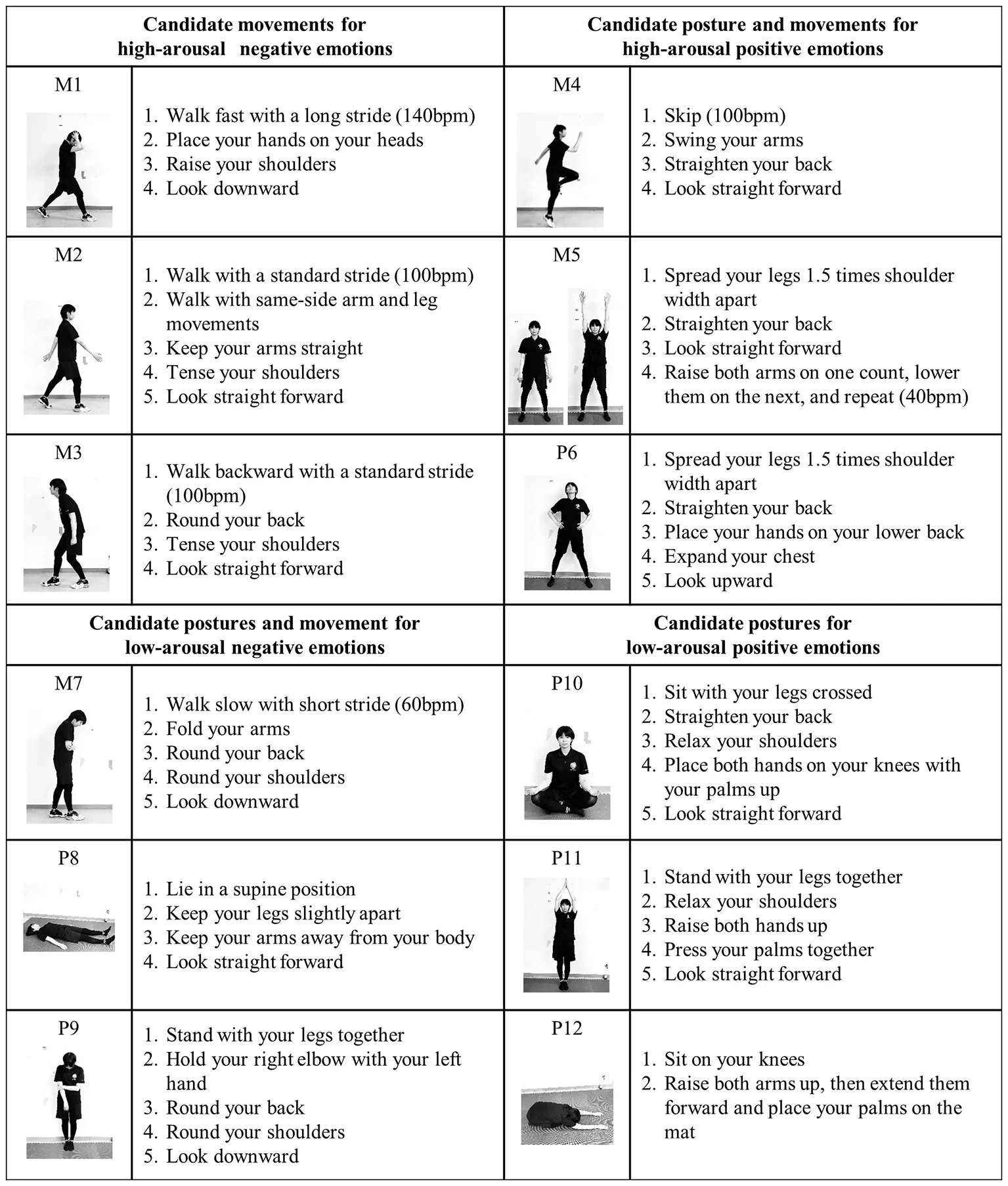
Instructions for the 12 candidate postures and movements in each category. M denotes movement; P denotes posture.

### 2.3 Measures

In this study, arousal and valence derived from the TDMS (Sakairi et al., 2013) were used as the primary psychological outcome measures. The TDMS was developed based on the two-dimensional theory of emotion and includes eight emotion words: “irritated” and “nervous” (high-arousal negative), “lively” and “energetic” (high-arousal positive), “lethargic” and “sluggish” (low-arousal negative), and “calm” and “relaxed” (low-arousal positive). Participants rated each item on a six-point Likert scale ranging from zero (“not at all”) to five (“very much”). First, the TDMS provides two subscale scores, activation and stability. Activation represents the contrast between high-arousal positive and low-arousal negative states and was calculated by subtracting the total score of the low-arousal negative items from that of the high-arousal positive items. Higher activation scores indicate a more energetic state, whereas lower scores indicate a more lethargic state. Stability represents the contrast between low-arousal positive and high-arousal negative states and was calculated by subtracting the total score of high-arousal negative items from that of low-arousal positive items. Higher stability scores indicate a calmer state, whereas lower scores indicate a more tense or irritated state. Both activation and stability range from −10 to +10. Arousal and valence were then calculated by mapping activation and stability onto the arousal and valence axes of the two-dimensional affective space. Arousal was calculated as activation minus stability, with higher scores indicating higher arousal and lower scores indicating lower arousal. Valence was calculated as activation plus stability, with higher scores indicating more positive valence and lower scores indicating more negative valence. Both arousal and valence range from −20 to +20. To examine psychological arousal and valence following each posture and movement, participants completed the TDMS after each condition. In addition to the quantitative measures, participants were asked to provide open-ended responses describing the emotions they experienced during each posture and movement. HR was also used to assess physiological arousal. HR was calculated from the R–R interval obtained using an HR monitor [Polar V800 (Polar Electro Oy, Kempele, Finland)] and analyzed using Kubios HRV Version 3.1 (Kubios Oy, Kuopio, Finland)(Tarvainen et al., 2014).

### 2.4 Procedure

The experiment was conducted over 2 days. Participants were instructed to perform either six postures or six movements per day. The order in which the posture and movement sessions were conducted (i.e., whether postures were performed on day 1 or day 2) was counterbalanced across participants. Within each session, the order of the six postures or movements was randomized. To minimize demand characteristics, participants were told a cover story in which the purpose of the study was to measure their physiological responses during various postures and movements. The participants were not informed that their posture and movement could affect psychological arousal or valence. Studies based on the facial feedback hypothesis (Coles et al., 2023) have demonstrated that bodily states can influence psychological states even in the absence of demand characteristics. By contrast, research on power posing has emphasized the importance of using cover stories to divert participants' attention from the true purpose of the experiment (Carney et al., 2015). Based on these considerations, we designed the cover story in this study with reference to previous research that attempted to minimize demand characteristics by presenting the participants with explanations akin to “measuring physiological responses while adopting postures” (e.g., Carney et al., 2010; Riskind and Gotay, 1982; Takayama and Sekiya, 2023). After participants entered the laboratory, the HR monitor transmitter was attached to their chest and recording was initiated. They were first instructed to adopt a seated upright posture for 2 min, during which they completed the TDMS to assess their psychological state in that posture. Following their responses, participants were verbally instructed to perform the first posture or movement for 1 min. After 1 min, they completed the TDMS and provided open-ended responses regarding their experiences during the posture or movement. This sequence, adopting a seated upright posture for 2 min, performing a posture or movement for 1 min, and completing the TDMS and providing open-ended responses, was repeated six times. At the end of the experimental day 2, the participants were debriefed regarding the cover story and the true purpose of the study was explained. A verbal check was also conducted to assess whether the demand characteristics were perceived.

### 2.5 Statistical analysis

Statistical analyses were conducted using R (Version 4.5.0; R Foundation for Statistical Computing, Vienna, Austria). The dplyr (Version 1.2.1) and tidyr (Version 1.3.2) packages were used for data processing, lme4 (Version 1.1-37) and lmerTest (Version 3.1-3) were used to estimate linear mixed-effects models, and emmeans (Version 2.0.4) was used to obtain estimated marginal means, conduct comparisons among conditions, and calculate model-based Cohen's *d*. Multilevel correlation analyses were conducted using HAD (version 18; Hiroshi Shimizu, Kwansei Gakuin University, Nishinomiya, Hyogo, Japan)(Shimizu, 2016), and morphological analysis of the open-ended responses was conducted using KH Coder (Koichi Higuchi, Ritsumeikan University, Kyoto City, Kyoto, Japan)(Higuchi, 2016, 2017). All statistical tests were two-tailed, with the significance level set at 5%.

#### 2.5.1 HR during inter-condition seated-upright periods and assessment of carryover effects

Prior to the primary analyses, HR during the 2-min seated-upright periods between conditions was analyzed to assess recovery before each condition and possible carryover effects from the preceding condition. Each 2-min period was divided into four consecutive 30-s phases, referred to as the first, second, third, and final phases. HR was calculated from the R–R interval data for each phase. R–R intervals between 0.30 and 2.00 s were considered physiologically plausible. A phase was excluded from the analysis if valid R–R intervals covered less than 80% of the phase, if it contained a data gap longer than 5 s, if the artifact rate exceeded 5%, or if no valid R–R interval data were available.

To account for between-participant differences in HR and the non-independence of the four phases obtained from the same 2-min seated-upright period, changes in HR over time were analyzed using a linear mixed-effects model with random intercepts for participant and for each 2-min seated-upright period. Phase, preceding condition, experimental day, trial order of the preceding condition, and session order were included as fixed effects. After testing the main effect of phase, all pairwise comparisons among the four phases were conducted, with Bonferroni correction applied to the six comparisons.

The mean HR during the last phase of each 2-min seated-upright period was then used as the HR immediately before the subsequent condition. The difference between this pre-condition HR and HR measured at the beginning of the session on that experimental day was calculated. This difference was used as the dependent variable in a linear mixed-effects model including the preceding condition, experimental day, trial order, and session order as fixed effects. To account for between-participant differences in HR and the non-independence of observations obtained from the same participant, a random intercept was specified for participant.

#### 2.5.2 Linear mixed-effects models for arousal and valence

To account for the non-independence of repeated observations arising from each participant completing the 12 posture and movement conditions, arousal and valence were analyzed separately using linear mixed-effects models. In the model with arousal as the dependent variable, posture and movement condition, experimental day, trial order within each experimental day, session order of the posture and movement sessions, and arousal at the beginning of the session were included as fixed effects. In the model with valence as the dependent variable, posture and movement condition, experimental day, trial order, session order, and valence at the beginning of the session were included as fixed effects.

Trial order was treated as a six-level categorical variable representing the first through sixth trials on each experimental day. Session order was treated as a two-level variable indicating whether the movement session or the posture session was conducted first. To account for between-participant differences in arousal and valence and the non-independence of observations obtained from the same participant, a random intercept was specified for participant in each model. Models were estimated using restricted maximum likelihood. Fixed effects were evaluated using Type III *F* tests with degrees of freedom approximated using the Satterthwaite method. Models were also examined for singular fit.

##### 2.5.2.1 Comparisons with the theoretical origin of the TDMS

As the primary analysis, estimated marginal means for each posture and movement condition obtained from the linear mixed-effects models were compared with zero to position arousal and valence following each condition within the two-dimensional affective space of the TDMS. Here, zero did not represent scores obtained at the beginning of the session or during the inter-condition seated-upright periods; rather, it represented the theoretical origin defined by the intersection of the arousal and valence axes of the TDMS. Thus, this analysis was not intended to determine whether scores changed from their pre-condition or seated-upright levels. Instead, it examined the direction in which the adjusted score following each condition was positioned relative to the theoretical origin of the TDMS.

To account for multiple testing, the 12 comparisons for arousal and the 12 comparisons for valence were treated as a single family of 24 comparisons, and Bonferroni corrections were applied to the *p*-values and 95% confidence intervals. Model-based Cohen's *d* was calculated for each condition. Cohen's *d* was calculated by dividing the difference between the adjusted estimated marginal mean for each condition and the theoretical origin of zero by the residual standard deviation of the corresponding linear mixed-effects model.

##### 2.5.2.2 Comparisons across posture and movement conditions

In addition to comparisons with the theoretical origin, relative differences among posture and movement conditions were examined. For arousal and valence, the main effect of posture and movement condition was first tested, and estimated marginal means for the 12 conditions were obtained from each model. All pairwise comparisons among the 12 conditions were then conducted separately for arousal and valence. The 66 comparisons for each outcome were treated as a single family, and Bonferroni corrections were applied to the *p* values and confidence intervals for the mean differences. Model-based Cohen's *d* was calculated for each pairwise comparison by dividing the adjusted mean difference between conditions by the residual standard deviation of the corresponding linear mixed-effects model.

#### 2.5.3 Analysis of HR across posture and movement conditions

Mean HR during each posture and movement condition was also analyzed using a linear mixed-effects model. Mean HR was entered as the dependent variable, and posture and movement condition, experimental day, trial order within each experimental day, session order of the posture and movement sessions, and HR at the beginning of the session were included as fixed effects. A random intercept was specified for participant to account for between-participant differences in HR and the non-independence of repeated observations obtained from the same participant.

Model estimation, tests of fixed effects, and calculation of estimated marginal means were conducted using the same procedures as those described for the arousal and valence models. After testing the main effect of posture and movement condition, all 66 pairwise comparisons among the 12 conditions were conducted. Bonferroni corrections were applied to the *p*-values and 95% confidence intervals for the mean differences. Model-based Cohen's *d* for each comparison was calculated by dividing the adjusted mean difference between conditions by the residual standard deviation of the model. Because HR does not have a theoretical origin analogous to that of the TDMS, HR was not compared with zero.

#### 2.5.4 Association between subjective arousal and HR

A multilevel correlation analysis was conducted to examine the association between subjective arousal and HR. Because the data had a hierarchical structure in which conditions (Level 1, within-person) were nested within participants (Level 2, between-person), within-person and between-person correlations were estimated separately. Prior to the analysis, intra-class correlation coefficients (ICCs) were calculated to quantify the degree of non-independence of the arousal and HR observations within participants.

An additional linear mixed-effects model was conducted to examine whether the association between subjective arousal and HR could be accounted for by differences among posture and movement conditions, including differences in physical demands. For HR, each participant's mean HR across the 12 conditions was calculated as the between-person component. The within-person component was calculated by subtracting each participant's mean HR from the HR observed during each condition. The between-person component was centered on the grand mean across participants. Subjective arousal was entered as the dependent variable, and the within-person HR component, between-person HR component, posture and movement condition, experimental day, trial order, session order, arousal at the beginning of the session, and HR at the beginning of the session were included as fixed effects. A random intercept was specified for participant to account for between-participant differences in subjective arousal and the non-independence of repeated observations obtained from the same participant.

#### 2.5.5 Analysis of open-ended responses

Open-ended responses describing emotions experienced during each posture and movement were subjected to morphological analysis using MeCab implemented in KH Coder (Higuchi, 2016, 2017). Words extracted through morphological analysis were matched against the Dictionary of Emotive Expressions (Nakamura, 1993), and emotion-related words included in the dictionary were extracted. “Relaxed,” which is included in the TDMS but not in the Dictionary of Emotive Expressions, was also included because it represented an emotional expression assessed in the present study. The frequencies of the extracted emotion-related words were then calculated for each posture and movement condition.

Because emotion-related words were classified using rule-based morphological analysis and dictionary matching rather than by human raters, inter-rater reliability was not applicable, and was therefore not calculated. The resulting frequencies represented the number of word occurrences rather than the number of participants who used each word; thus, when the same participant used the same word multiple times, each occurrence was counted separately. The analysis of open-ended responses was treated as a supplementary exploratory analysis intended to descriptively identify emotional expressions that may not be readily captured by the TDMS.

## 3 Results

### 3.1 HR during inter-condition seated-upright periods and assessment of carryover effects

Of the 960 phases, 47 were excluded: 33 were excluded because valid R–R intervals covered less than 80% of the phase, 13 because a data gap longer than 5 s was present and one because no valid R–R interval data were available. Thus, 913 phases were included in the analysis. Of these, 760 phases obtained during the inter-condition seated-upright periods were used to analyze temporal changes in HR. After adjusting for the preceding condition, experimental day, trial order, and session order, the main effect of phase was significant [*F*_(3, 558.82)_ = 8.88, *p* < 0.001]. HR during the last phase was significantly higher than during the preceding three phases (adjusted *ps* < 0.009), with small differences ranging from 1.00 to 1.51 bpm. Because participants completed the TDMS and open-ended responses after each condition and before the subsequent seated-upright period, these four phases did not represent a continuous HR recovery trajectory beginning immediately after completion of the preceding condition.

The adjusted mean difference between HR immediately before each condition and HR at the beginning of the session was −0.74 bpm and was not significant [95% CI [−2.67, 1.20], *t*_(16.67)_ = −0.81, *p* = 0.431]. Neither the effect of the preceding condition nor that of trial order was significant [preceding condition: *F*_(11, 146.75)_ = 0.46, *p* = 0.928; trial order: *F*_(1, 145.34)_ = 0.07, *p* = 0.797]. Thus, no clear HR carryover effect attributable to a specific posture or movement condition was observed.

### 3.2 Linear mixed-effects models for arousal and valence

Each model for arousal and valence included 240 observations from 20 participants. For arousal, the main effect of posture and movement condition was significant [*F*_(11, 197.28)_ = 31.20, *p* < 0.001]. The effects of experimental day, trial order, session order, and arousal at the beginning of the session were not significant (*ps* ≥ 0.319). For valence, the main effect of posture and movement condition was also significant [*F*_(11, 202.62)_ = 8.66, *p* < 0.001]. The effects of experimental day, trial order, session order, and valence at the beginning of the session were not significant (*ps* ≥ 0.068).

#### 3.2.1 Comparisons with the theoretical origin of the TDMS

The adjusted estimated marginal means, 95% confidence intervals, comparisons with the theoretical origin, and Cohen's *d* values for each condition are presented in Table 1. The adjusted estimated marginal means and 95% confidence intervals for arousal and valence are also outlined in Figure 2. Bonferroni corrections were applied to the *p*-values and 95% confidence intervals across the 24 comparisons, comprising 12 comparisons for arousal and 12 for valence.

**Table 1:** Adjusted estimated marginal means and comparisons with the theoretical origin for arousal and valence.

Condition	Adjusted estimate	SE	95% CI	*t*	Adjusted *p*	Cohen's *d*
Arousal						
M1	6.76	1.26	[2.82, 10.70]	5.38	< 0.001	1.34
M2	2.23	1.25	[−1.69, 6.15]	1.78	1.000	0.44
M3	1.58	1.26	[−2.36, 5.53]	1.26	1.000	0.31
M4	6.95	1.27	[2.96, 10.94]	5.46	< 0.001	1.37
M5	−2.92	1.26	[−6.87, 1.04]	−2.31	0.534	−0.58
P6	−0.72	1.26	[−4.66, 3.22]	−0.57	1.000	−0.14
M7	−8.00	1.26	[−11.96, −4.04]	−6.33	< 0.001	−1.58
P8	−13.77	1.25	[−17.69, −9.85]	−11.00	< 0.001	−2.72
P9	−6.76	1.25	[−10.67, −2.85]	−5.42	< 0.001	−1.34
P10	−7.32	1.25	[−11.23, −3.40]	−5.86	< 0.001	−1.45
P11	1.78	1.27	[−2.20, 5.76]	1.40	1.000	0.35
P12	−6.82	1.27	[−10.79, −2.84]	−5.37	< 0.001	−1.35
Valence						
M1	2.20	0.96	[−0.79, 5.19]	2.29	0.551	0.55
M2	1.40	0.95	[−1.58, 4.37]	1.46	1.000	0.35
M3	−0.91	0.96	[−3.91, 2.09]	−0.95	1.000	−0.23
M4	7.99	0.97	[4.95, 11.02]	8.21	< 0.001	1.98
M5	2.95	0.96	[−0.06, 5.96]	3.06	0.060	0.73
P6	6.03	0.96	[3.04, 9.03]	6.29	< 0.001	1.50
M7	0.08	0.96	[−2.93, 3.09]	0.08	1.000	0.02
P8	3.41	0.95	[0.44, 6.39]	3.58	0.011	0.85
P9	−1.41	0.95	[−4.37, 1.56]	−1.48	1.000	−0.35
P10	3.45	0.95	[0.48, 6.43]	3.63	0.009	0.86
P11	1.14	0.97	[−1.89, 4.16]	1.17	1.000	0.28
P12	1.18	0.97	[−1.85, 4.20]	1.21	1.000	0.29

Values are estimated marginal means obtained from linear mixed-effects models adjusted for experimental day, trial order, session order, and session-baseline scores. The *p*-values and 95% confidence intervals were adjusted using the Bonferroni method across the 24 comparisons comprising 12 arousal and 12 valence comparisons. Cohen's *d* values were calculated by dividing each estimated marginal mean by the residual standard deviation of the corresponding model. M denotes movement; P denotes posture.

**Figure 2:**
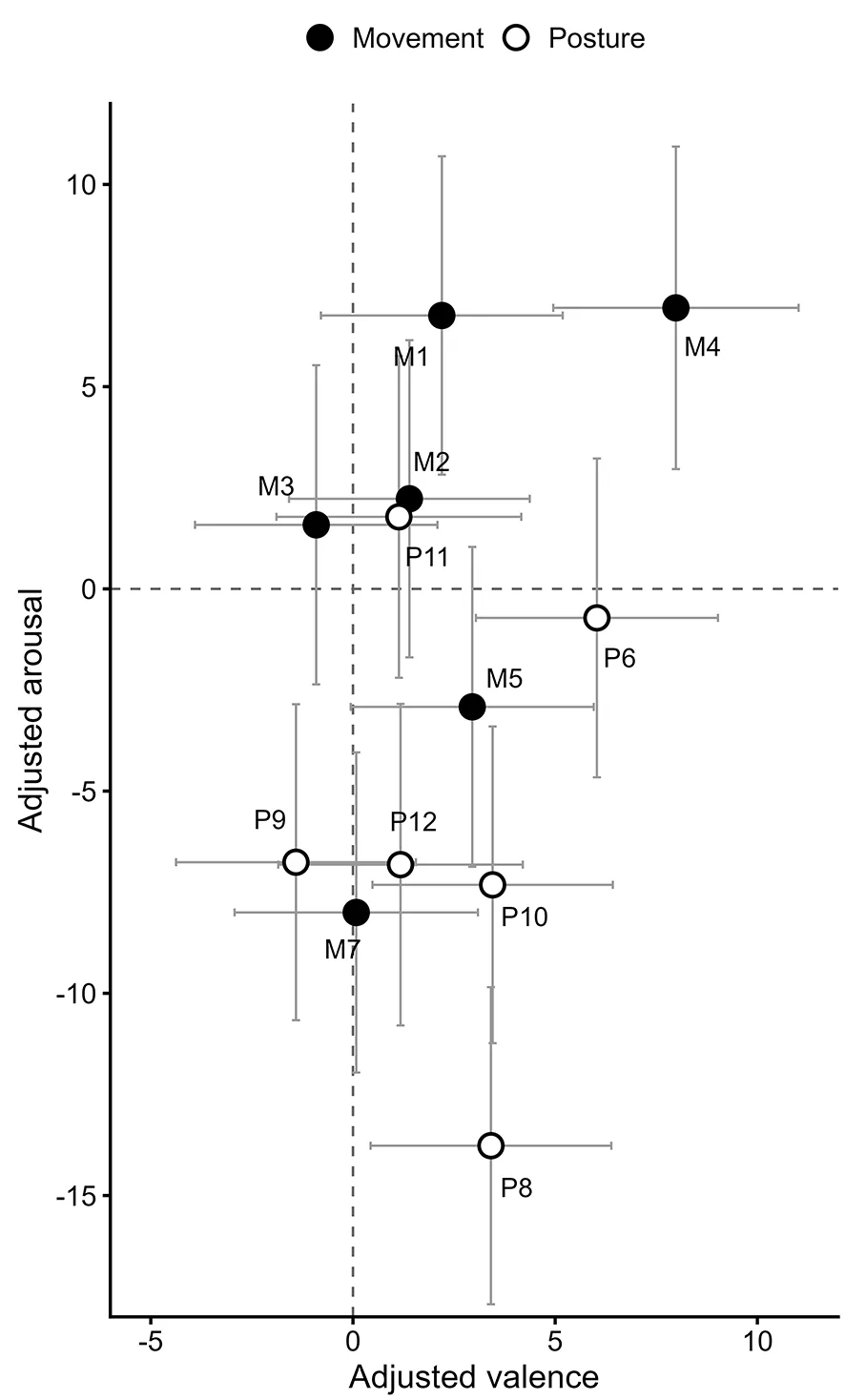
Adjusted estimated marginal means for arousal and valence across posture and movement conditions. Points represent estimated marginal means obtained from linear mixed-effects models adjusted for experimental day, trial order, session order, and session-baseline scores. Horizontal and vertical error bars represent 95% confidence intervals for valence and arousal, respectively. The confidence intervals were adjusted using the Bonferroni method across the 24 comparisons comprising 12 arousal and 12 valence comparisons. Filled circles indicate movement conditions, and open circles indicate posture conditions. Dashed lines indicate the theoretical origin of the TDMS affective space. M denotes movement, and P denotes posture.

For arousal, the adjusted estimated marginal means for M1 and M4 were significantly higher than the theoretical origin [M1: estimate = 6.76, 95% CI [2.82, 10.70], adjusted *p* < 0.001, Cohen's *d* = 1.34; M4: estimate = 6.95, 95% CI [2.96, 10.94], adjusted *p* < 0.001, Cohen's *d* = 1.37]. By contrast, M7, P8, P9, P10, and P12 were significantly lower than the theoretical origin (adjusted *ps* < 0.001). Among these conditions, P8 exhibited the lowest value [estimate = −13.77, 95% CI [−17.69, −9.85], adjusted *p* < 0.001, Cohen's *d* = −2.72]. For M2, M3, M5, P6, and P11, the differences from the theoretical origin were not significant after Bonferroni correction.

For valence, the adjusted estimated marginal means for M4, P6, P8, and P10 were significantly higher than the theoretical origin [M4: estimate = 7.99, 95% CI [4.95, 11.02], adjusted *p* < 0.001, Cohen's *d* = 1.98; P6: estimate = 6.03, 95% CI [3.04, 9.03], adjusted *p* < 0.001, Cohen's *d* = 1.50; P8: estimate = 3.41, 95% CI [0.44, 6.39], adjusted *p* = 0.011, Cohen's *d* = 0.85; P10: estimate = 3.45, 95% CI [0.48, 6.43], adjusted *p* = 0.009, Cohen's *d* = 0.86]. For the remaining conditions, differences from the theoretical origin were not significant after Bonferroni correction. No condition demonstrated valence that was significantly lower than the theoretical origin.

#### 3.2.2 Comparisons across posture and movement conditions

For arousal, no significant difference was observed between M1 and M4. M1 and M4 showed significantly higher adjusted arousal than M5, M7, P6, P8, P9, P10, and P12 (adjusted *ps* < 0.05). By contrast, no significant differences were observed after Bonferroni correction between M1 or M4 and M2, M3, or P11. P8 revealed the lowest adjusted arousal and was significantly lower than multiple posture and movement conditions. The remaining pairwise comparisons are presented in Supplementary Table S1.

For valence, M4 showed significantly higher adjusted valence than M1, M2, M3, M5, M7, P8, P9, P10, P11, and P12 (adjusted *ps* < 0.05). By contrast, no significant difference was observed between M4 and P6. P6 showed significantly higher adjusted valence than M2, M3, M7, P9, P11, and P12 (adjusted *ps* < 0.05). The remaining pairwise comparisons are presented in Supplementary Table S2.

### 3.3 HR across posture and movement conditions

For mean HR during each condition, the main effect of posture and movement condition was significant [*F*_(11, 204.58)_ = 77.30, *p* < 0.001]. The effects of session order [*F*_(1, 17.52)_ = 8.37, *p* = 0.010] and HR at the beginning of the session [*F*_(1, 47.05)_ = 72.74, *p* < 0.001] were also significant. By contrast, the effects of experimental day and trial order were not significant (*ps* ≥ 0.466). The two highest adjusted estimated marginal mean HR values were observed for M4 (118.35 bpm, 95% CI [114.19, 122.52]) and M1 (116.32 bpm, 95% CI [112.21, 120.43]) and lowest observed for P8 (63.78 bpm, 95% CI [59.69, 67.87]). Detailed pairwise comparisons among the 12 conditions are presented in Supplementary Table S3.

### 3.4 Association between subjective arousal and HR

To analyze the relationship between subjective and objective arousal, we first computed ICCs for subjective arousal (TDMS arousal score) and HR. The ICC for subjective arousal was small and not statistically different from zero (ICC = 0.02, *p* = 0.21), whereas the ICC for HR was significant (ICC = 0.13, *p* < 0.001), indicating non-independence of observations. Therefore, we conducted a multilevel correlation analysis to account for the nested data structure, with posture/movement conditions (Level 1, within-person) nested within individuals (Level 2, between-person). The results revealed a significant positive correlation at the within-person level (*r* = 0.67, *p* < 0.001), indicating that, across posture and movement conditions, conditions with higher HR tended to be associated with higher subjective arousal in the same participant.

Following this, we conducted a linear mixed-effects model to examine whether this association could be explained by differences among posture and movement conditions. The model adjusted for posture and movement condition, experimental day, trial order, session order, arousal at the beginning of the session, and HR at the beginning of the session. The within-person HR component was not significantly associated with subjective arousal [*B* = 0.053, *SE* = 0.041, 95% CI [−0.028, 0.134], *t*_(210.32)_ = 1.29, *p* = 0.198]. By contrast, the between-person HR component was significantly associated with subjective arousal [*B* = 0.260, *SE* = 0.096, 95% CI [0.072, 0.448], *t*_(21.92)_ = 2.71, *p* = 0.013].

### 3.5 Emotion-related expressions identified from open-ended responses

Morphological analysis was conducted on the open-ended responses describing emotions experienced during each posture and movement. The corpus contained 2,066 total word occurrences and 405 unique words. Emotion-related words extracted from the open-ended responses for each condition using the Dictionary of Emotive Expressions, together with their frequencies, are presented in Table 2.

**Table 2:** Emotion-related words and expressions identified for each posture and movement through morphological analysis of open-ended responses.

Condition	Extracted word	Frequency	Condition	Extracted word	Frequency
M1	Restless	3	M7	Dark	2
	Nervous^a^	1		Suffocating	1
	Calm^a^	1		Lethargic^a^	1
				Calm^a^	1
M2	Strain	2		Relaxed^a^	1
	Dislike	1			
	Stiff	1	P8	Relaxed^a^	5
	Laugh	1		Comfortable	3
	Lethargic^a^ (negated)	1		Pleasant	1
				Calm^a^	1
M3	Strain	2			
	Scared	1	P9	Dark	2
	Fear	1		Anxious	1
	Anxious	1		Awkward	1
	Like (negated)	1		Lonely	1
	Stiff	1		Painful	1
	Nervous^a^	1		Sad	1
				Lethargic^a^	1
M4	Fun	5			
	Smiling	1	P10	Painful	2
	Irritated^a^ (negated)	1		Relaxed^a^	2
	Anxious	1		Comfortable	1
	Strain	1		Calm^a^ (negated)	1
	Nervous ^a^	1			
			P11	Calm ^a^	1
M5	Strain	2		Strain	1
	Calm^a^	2		Nauseated	1
	Comfortable	1		Relaxed^a^ (negated)	1
	Angry	1			
	Irritated^a^	1	P12	Pleasurable	7
				Calm^a^	1
P6	Pleasurable	2		Relaxed^a^	1
	Refreshed	2		Miserable	1
	Bright	1		Suffocating	1
	Relaxed^a^	1		Relaxed^a^ (negated)	1
	Comfortable (negated)	1			
	Painful	1			

M denotes movement; P denotes posture. Negated denotes extracted word negated in the original response. Frequencies represent word occurrences, not the number of participants who used each word. Repeated use of the same word by a participant was counted each time. “Relaxed,” which is included in the TDMS but not in the Dictionary of Emotive Expressions, was additionally included in the analysis.

^a^Indicates that the word was included as an item on the Two-Dimensional Mood Scale.

The open-ended responses included words corresponding to TDMS items, such as “calm,” “irritated,” “lethargic,” “relaxed,” and “nervous.” Words not directly included among the TDMS items were also extracted, including “restless,” “strain,” “scared,” “fear,” “anxious,” “fun,” “smiling,” “pleasurable,” “refreshed,” “dark,” “painful,” “suffocating,” and “comfortable.” By condition, “restless” was obtained for M1. For M2, words such as “strain,” “relaxed,” and “dislike” were noted. For M3, “scared,” “fear,” and “anxious” were observed in addition to “nervous.” For M4, expressions related to positive affect, such as “fun” and “smiling,” were observed. For M5, both “strain” and “calm” were noted. For P6, “pleasurable” and “refreshed” were recorded. “Dark” was obtained for M7 and P9. For P8, “relaxed” and “comfortable” were discerned. For P10, both “painful” and “relaxed” were noted. For P11, “strain” occurred in addition to “calm.” For P12, “pleasurable” was noted.

These findings were exploratory and based on the frequency of word occurrences in the open-ended responses; they do not represent the number of participants who used each word. In addition, some words occurred only once or a few times. Therefore, these findings should not be interpreted as indicating that particular emotions were shared across participants within a condition.

## 4 Discussion

The primary objective of this study was to exploratorily examine psychological arousal and valence following different postures and movements. Accordingly, we examined their relationships with the theoretical origin of the two-dimensional affective space of the TDMS. With respect to psychological arousal, M1 (fast walking) and M4 (skipping) were associated with adjusted arousal scores that were significantly higher than the theoretical origin. Moreover, M1 and M4 showed relatively high adjusted mean HR among the 12 posture and movement conditions [M1: 116.32 bpm, 95% CI [112.21, 120.43]; M4: 118.35 bpm, 95% CI [114.19, 122.52]]. Thus, the relatively high physiological activity and physical demands accompanying these movements may have been associated with higher subjective arousal. M2, M3, and M4 were all performed at 100 bpm; however, after Bonferroni correction, only M4 exhibited significantly higher adjusted arousal than the theoretical origin, whereas M2 and M3 were not positioned in the high-arousal region. In addition, the adjusted mean HR for M4 was significantly higher than those for M2 and M3. These findings suggest that differences in psychological arousal cannot be explained solely by movement tempo. Rather, the vertical bodily movement and greater whole-body activity involved in skipping may have been associated with both higher HR and higher subjective arousal. By contrast, M5, which involved raising the arms while standing, was not positioned in the high-arousal region. Because M5 was performed at a slow tempo of 40 bpm, the overall level of physical activity may have remained limited despite the arm-raising movement.

The present study included not only movements but also static postures to explore their positioning within the two-dimensional affective space of the TDMS. Therefore, P6, in which participants placed their hands on the lower back and expanded the chest, was included as a posture potentially associated with high arousal. P6 was selected with reference to high-power poses; however, its adjusted arousal did not differ significantly from the theoretical origin, leaving its position on the arousal axis unclear. Furthermore, adjusted arousal for P6 was significantly lower than that for M1 and M4. Thus, at least for P6 as implemented in the present study, arousal comparable to that associated with fast walking or skipping was not observed. This finding is consistent with Takayama and Sekiya (2023), who suggested that when individuals are instructed to adopt a specific standing posture, muscle activity outside the lower limbs may decrease, potentially limiting the association between standing posture and arousal.

Among M7 and P8–P12, which were selected as candidate low-arousal conditions, five conditions other than P11 exhibited significantly lower adjusted arousal than the theoretical origin. Notably, M7 was positioned in the low-arousal region despite being a movement condition. M7 consisted of walking at a slow tempo of 60 bpm, and this finding is consistent with previous research indicating that slow-tempo movements are less likely to increase psychological arousal (Naruse and Hirai, 2000). Therefore, the present findings indicate that low arousal may be associated not only with static postures but also with slow-tempo walking.

By contrast, adjusted arousal for P11 did not differ significantly from the theoretical origin, leaving its position on the arousal axis unclear. Adjusted arousal for P11 was also significantly higher than that for P8, P9, P10, and P12. Because P11 required participants to maintain a posture with the arms raised, it may have imposed greater physical demands than the other candidate low-arousal postures. Indeed, the adjusted mean HR for P11 was significantly higher than those for P8, P10, and P12, suggesting that the physical demands associated with maintaining the arms in a raised position may have contributed to P11 not being positioned in the low-arousal region.

With respect to valence, M4, P6, P8, and P10 showed adjusted valence scores that were significantly higher than the theoretical origin. M4 (skipping) was selected based on Peper and Lin (2012), who noted that skipping may be associated with high-arousal positive emotion. In the present study, M4 was positioned in the high-arousal positive quadrant, and its adjusted valence was significantly higher than that of all other conditions except P6. P6, which involved placing the hands on the lower back and expanding the chest, was selected with reference to high-power poses (Carney et al., 2010) and was positioned in the positive direction. Because no significant difference in adjusted valence was observed between M4 and P6, not only movements such as skipping but also expansive postures involving an open chest may be associated with relatively high valence.

Among P10–P12, which were selected as candidate low-arousal positive conditions, P10 (meditation posture) was positioned in the low-arousal positive quadrant. By contrast, adjusted valence for P11 and P12 did not differ significantly from the theoretical origin, rendering their positions on the valence axis unclear. The finding for P10 suggests that even a brief meditation posture without controlled breathing or attention may be associated with a combination of low arousal and positive valence, consistent with Takayama and Sekiya (2023). P8 (supine posture), which had been selected as a candidate low-arousal negative condition, was unexpectedly positioned in the low-arousal positive quadrant. We had expected that sleepiness associated with the supine posture would be related to feelings of lethargy or sluggishness. However, the present results indicate that adopting the supine posture for 1 min was associated with a low-arousal positive rather than negative state. Thus, low arousal is not necessarily accompanied by negative valence; when the body is placed in a supine position, low arousal may instead be experienced together with positive valence.

No posture or movement condition demonstrated adjusted valence that was significantly lower than the theoretical origin. Many of the conditions selected as candidates for low-arousal negative emotion either had an unclear position on the valence axis or, as in the case of P8, were positioned in the positive direction. These findings indicate that postures and movements associated with low arousal are not necessarily positioned in the negative direction. An important finding of the present study is that evaluating arousal and valence as two independent dimensions made it possible to demonstrate that states characterized by similarly low arousal can differ in their positioning along the valence dimension.

In addition to words corresponding to TDMS items, the open-ended responses included expressions related to fear and anxiety, such as “scared,” “fear,” and “anxious”; positive experiences, such as “fun” and “smiling”; pain, such as “painful”; and bodily discomfort, such as “strain,” “suffocating,” “stiff,” and “nauseated.” These findings suggest that subjective experiences that may not be readily captured by the TDMS can be described in open-ended responses. However, the extracted results represent frequencies of word occurrences rather than the number of participants who used each word, and many words occurred only infrequently. Therefore, the open-ended responses should not be interpreted as evidence that particular emotions were shared across participants or elicited by specific conditions. Rather, they should be regarded as supplementary exploratory findings that complement the TDMS and identify emotional expressions and bodily sensations that may warrant further investigation.

The second aim of the present study was to examine the relationship between HR during each posture and movement condition and subjective arousal rated after each condition. The multilevel correlation analysis examined covariation between HR and subjective arousal across the 12 conditions performed by each participant while accounting for the nested structure of the repeated observations. Conditions with relatively higher HR tended to be rated as higher in subjective arousal in the same participant. This finding is consistent with previous research showing a positive association between psychological arousal and HR (Kreibig, 2010; Takayama and Sekiya, 2023). However, this analysis also included differences in physical demands across conditions, such as those associated with fast walking and skipping. Therefore, we conducted an additional linear mixed-effects model to examine the association between within-person variation in HR and subjective arousal after statistically adjusting for posture and movement condition, experimental day, trial order, session order, and states at the beginning of the session. The within-person HR component was not significantly associated with subjective arousal. Thus, after adjustment for posture and movement condition and the other covariates, no evidence was found to support an association between condition-independent within-person variation in HR and subjective arousal. Accordingly, the positive within-person association observed in the multilevel correlation analysis may not have reflected condition-independent HR variation alone, but may instead have reflected covariation that included systematic differences among the posture and movement conditions. By contrast, the between-person HR component was significantly associated with subjective arousal, indicating that participants with higher mean HR tended to report higher subjective arousal overall.

Within the framework of embodiment theory, physiological changes, including autonomic nervous system activity accompanying bodily activity, can be regarded as part of the bodily information associated with emotional experience. In the present study, some movement conditions, particularly M1 (fast walking) and M4 (skipping), showed relatively high subjective arousal and HR, and M4 was also positioned in the high-arousal positive region. However, the linear mixed-effects model adjusting for posture and movement condition and the other covariates did not exhibit an independent association between within-person variation in HR and subjective arousal. Therefore, these findings should not be interpreted as evidence that increased HR itself heightened subjective arousal. Rather, physical characteristics of fast walking and skipping, such as the amount of bodily activity, movement speed, and vertical bodily motion, may have been associated with both HR and subjective arousal. Previous research has also reported that walking speed and vertical bodily motion during walking may be associated with emotional states (Adolph et al., 2021). Taken together, the present findings suggest that understanding the correspondence between physiological and subjective arousal accompanying bodily activity requires consideration not only of a single physiological indicator, such as HR, but also of the physical characteristics that constitute each posture and movement.

### 4.1 Limitations

This study has a few limitations. First, the sample was limited to 20 university students, which restricts the generalizability of the findings. In addition, the *a priori* power analysis conducted during study planning was based on effect sizes that had reached statistical significance in previous research and assumed a single one-sample test against the theoretical origin. Therefore, it did not ensure sufficient statistical power for comparisons after Bonferroni correction or for comparisons among conditions using linear mixed-effects models. Accordingly, the present findings should be interpreted cautiously as exploratory evidence observed immediately following brief posture and movement conditions.

Second, although a 2-min seated-upright period was provided between conditions, whether subjective and physiological states fully recovered within this period cannot be definitively concluded. HR immediately before each condition did not differ significantly from HR at the beginning of the session, and no significant effects of the preceding condition or trial order were observed, implying that no clear carryover effect on HR was detected. However, small differences in HR were observed across the four phases of the 2-min seated-upright period. In addition, because the TDMS was not administered immediately before each condition, determining whether subjective arousal and valence had returned to their levels at the beginning of the session was not possible.

Third, exercise intensity, movement form, movement tempo, and vertical bodily movement were not manipulated independently in the present study. Therefore, for conditions such as M4, wherein both HR and subjective arousal were high, identifying which specific bodily component was associated with the observed results was not possible. Physiological changes accompanying movement can be regarded as one pathway involved in embodied emotional experience; however, the present findings cannot disentangle the physiological effects of exercise intensity from the effects of movement form, rhythm, and psychological meaning.

Fourth, because a metronome was used to standardize movement tempo, its auditory stimulation may have influenced participants' emotional responses. Research on music has shown that faster tempos are associated with increased arousal, whereas slower tempos are associated with decreased arousal. Thus, emotional states may have been associated not only with the movements themselves but also with the auditory perception of tempo. Future studies should consider alternative procedures, such as providing verbal instructions regarding tempo or presenting the metronome only briefly before movement execution and having participants perform the movements in silence.

Fifth, individual differences in habitual posture and walking speed may have influenced perceived physical demands. For example, some participants reported that the fast-walking condition felt similar to their usual walking speed, whereas others reported habitually adopting poor posture. Such individual differences may have influenced the associations between each condition and emotional state. Future studies should assess participants' baseline postural and walking characteristics and examine how deviations from their habitual states are associated with emotional responses.

## 5 Conclusion

This study examined psychological arousal, valence, and HR following 12 different postures and movements. Comparisons with the theoretical origin of the two-dimensional affective space of the TDMS positioned skipping in the high-arousal positive quadrant, whereas the supine and meditation postures were positioned in the low-arousal positive quadrant. Comparisons among conditions showed that fast walking and skipping were associated with higher arousal than several other posture and movement conditions, whereas skipping and the expansive chest posture were associated with higher valence than several other conditions. HR was relatively high during fast walking and skipping, and lowest during the supine posture. Furthermore, subjective arousal was positively associated with HR at the within-person level after accounting for between-person differences. However, this within-person association was not significant in an additional linear mixed-effects model adjusting for posture and movement condition and other covariates. Thus, the association between HR and subjective arousal may have reflected differences in bodily states across posture and movement conditions, including differences in physical demands, rather than HR fluctuations occurring independently of condition. Overall, the present study provides exploratory evidence regarding the positioning of emotional states following different postures and movements within a two-dimensional model of emotion and suggests that differences in bodily states across posture and movement conditions may contribute to the relationship between subjective arousal and HR.

## Data Availability

The datasets presented in this study can be found in online repositories. The names of the repository/repositories and accession number(s) can be found below: Open Science Framework (OSF): https://osf.io/jehpr/.
