# Supplementary material for: Investigating postures and movements associated with psychological arousal and valence
Source: Front Psychol. 2026 Sep 14;17:1866036. doi: 10.3389/fpsyg.2026.1866036 (PMC13616642; doi:10.3389/fpsyg.2026.1866036)
Supplement: Supplementary file 1 [file Table_1.pdf]

Supplementary Material

1 Supplementary Table S1 Pairwise comparisons of adjusted estimated marginal means for arousal across conditions

Comparison	Adjusted mean difference	SE	Bonferroni-adjusted 95% CI	t	Adjusted *p*	Cohen's *d*
M1 - M2	4.53	1.62	[-1.01, 10.08]	2.80	.375	0.90
M1 - M3	5.18	1.62	[-0.38, 10.73]	3.19	.109	1.02
M1 - M4	-0.19	1.62	[-5.75, 5.37]	-0.12	1.000	-0.04
M1 - M5	9.68	1.63	[4.11, 15.25]	5.94	< .001	1.91
M1 - M7	14.76	1.64	[9.16, 20.36]	9.01	< .001	2.92
M1 - P6	7.48	1.62	[1.95, 13.01]	4.63	< .001	1.48
M1 - P8	20.53	1.61	[15.02, 26.04]	12.75	< .001	4.06
M1 - P9	13.52	1.61	[8.00, 19.04]	8.38	< .001	2.67
M1 - P10	14.08	1.62	[8.54, 19.62]	8.70	< .001	2.78
M1 - P11	4.98	1.62	[-0.56, 10.51]	3.08	.157	0.98
M1 - P12	13.58	1.64	[7.96, 19.19]	8.27	< .001	2.68
M2 - M3	0.64	1.63	[-4.92, 6.21]	0.40	1.000	0.13
M2 - M4	-4.72	1.63	[-10.31, 0.87]	-2.89	.284	-0.93
M2 - M5	5.14	1.62	[-0.40, 10.69]	3.17	.115	1.02
M2 - M7	10.23	1.61	[4.74, 15.72]	6.37	< .001	2.02
M2 - P6	2.95	1.61	[-2.57, 8.46]	1.83	1.000	0.58
M2 - P8	16.00	1.61	[10.49, 21.50]	9.94	< .001	3.16
M2 - P9	8.99	1.61	[3.49, 14.49]	5.59	< .001	1.78
M2 - P10	9.55	1.61	[4.03, 15.06]	5.93	< .001	1.89
M2 - P11	0.45	1.64	[-5.16, 6.05]	0.27	1.000	0.09
M2 - P12	9.05	1.62	[3.52, 14.58]	5.60	< .001	1.79
M3 - M4	-5.36	1.65	[-11.00, 0.27]	-3.25	.088	-1.06
M3 - M5	4.50	1.61	[-1.02, 10.02]	2.79	.382	0.89
M3 - M7	9.59	1.64	[3.98, 15.19]	5.85	< .001	1.89
M3 - P6	2.30	1.64	[-3.29, 7.90]	1.41	1.000	0.45
M3 - P8	15.35	1.63	[9.78, 20.92]	9.43	< .001	3.03
M3 - P9	8.34	1.61	[2.84, 13.85]	5.18	< .001	1.65
M3 - P10	8.90	1.61	[3.40, 14.40]	5.54	< .001	1.76
M3 - P11	-0.20	1.61	[-5.72, 5.32]	-0.12	1.000	-0.04
M3 - P12	8.40	1.64	[2.80, 14.01]	5.13	< .001	1.66
M4 - M5	9.87	1.66	[4.19, 15.54]	5.95	< .001	1.95
M4 - M7	14.95	1.64	[9.32, 20.58]	9.09	< .001	2.95
M4 - P6	7.67	1.63	[2.10, 13.23]	4.71	< .001	1.51
M4 - P8	20.72	1.61	[15.21, 26.23]	12.86	< .001	4.09
M4 - P9	13.71	1.63	[8.12, 19.29]	8.39	< .001	2.71
M4 - P10	14.27	1.63	[8.70, 19.83]	8.77	< .001	2.82
M4 - P11	5.17	1.65	[-0.46, 10.80]	3.14	.129	1.02
M4 - P12	13.77	1.65	[8.12, 19.42]	8.33	< .001	2.72

Comparison	Adjusted mean difference	SE	Bonferroni-adjusted 95% CI	t	Adjusted *p*	Cohen's *d*
M5 - M7	5.08	1.64	[-0.53, 10.70]	3.10	.146	1.00
M5 - P6	-2.20	1.64	[-7.79, 3.40]	-1.34	1.000	-0.43
M5 - P8	10.85	1.63	[5.26, 16.44]	6.64	< .001	2.14
M5 - P9	3.84	1.61	[-1.66, 9.34]	2.39	1.000	0.76
M5 - P10	4.40	1.62	[-1.14, 9.95]	2.72	.476	0.87
M5 - P11	-4.70	1.64	[-10.29, 0.90]	-2.87	.298	-0.93
M5 - P12	3.90	1.61	[-1.61, 9.42]	2.42	1.000	0.77
M7 - P6	-7.28	1.61	[-12.81, -1.76]	-4.51	< .001	-1.44
M7 - P8	5.77	1.62	[0.24, 11.30]	3.57	.030	1.14
M7 - P9	-1.24	1.62	[-6.78, 4.29]	-0.77	1.000	-0.25
M7 - P10	-0.68	1.62	[-6.21, 4.84]	-0.42	1.000	-0.14
M7 - P11	-9.78	1.65	[-15.43, -4.13]	-5.92	< .001	-1.93
M7 - P12	-1.18	1.63	[-6.77, 4.40]	-0.72	1.000	-0.23
P6 - P8	13.05	1.61	[7.55, 18.55]	8.11	< .001	2.58
P6 - P9	6.04	1.61	[0.53, 11.56]	3.75	.016	1.19
P6 - P10	6.60	1.62	[1.06, 12.14]	4.07	.004	1.30
P6 - P11	-2.50	1.65	[-8.14, 3.14]	-1.52	1.000	-0.49
P6 - P12	6.10	1.63	[0.51, 11.69]	3.74	.016	1.21
P8 - P9	-7.01	1.61	[-12.53, -1.49]	-4.35	.001	-1.39
P8 - P10	-6.45	1.61	[-11.96, -0.94]	-4.00	.006	-1.27
P8 - P11	-15.55	1.63	[-21.12, -9.98]	-9.55	< .001	-3.07
P8 - P12	-6.95	1.63	[-12.53, -1.37]	-4.26	.002	-1.37
P9 - P10	0.56	1.61	[-4.94, 6.06]	0.35	1.000	0.11
P9 - P11	-8.54	1.63	[-14.10, -2.98]	-5.25	< .001	-1.69
P9 - P12	0.06	1.62	[-5.47, 5.59]	0.04	1.000	0.01
P10 - P11	-9.10	1.62	[-14.63, -3.57]	-5.63	< .001	-1.80
P10 - P12	-0.50	1.63	[-6.08, 5.08]	-0.31	1.000	-0.10
P11 - P12	8.60	1.66	[2.91, 14.29]	5.17	< .001	1.70

Note. Pairwise comparisons were based on estimated marginal means obtained from the linear mixed-effects model adjusted for experimental day, trial order, session order, and session-baseline arousal. The p values and confidence intervals were adjusted using the Bonferroni method across all 66 pairwise comparisons. Degrees of freedom were estimated using the Satterthwaite method and were 197.28 for all comparisons. Standardized effects were calculated by dividing each adjusted mean difference by the residual standard deviation of the model. A negative value indicates that the first condition had a lower estimated arousal score than the second condition. M denotes movement; P denotes posture.

Supplementary Table S2

Pairwise comparisons of adjusted estimated marginal means for valence across conditions

Comparison	Adjusted mean difference	SE	Bonferroni-adjusted 95% CI	t	Adjusted *p*	Cohen's *d*
M1 - M2	0.80	1.29	[-3.61, 5.21]	0.62	1.000	0.20
M1 - M3	3.11	1.29	[-1.31, 7.52]	2.41	1.000	0.77
M1 - M4	-5.79	1.29	[-10.21, -1.37]	-4.48	< .001	-1.44
M1 - M5	-0.75	1.29	[-5.18, 3.67]	-0.58	1.000	-0.19
M1 - M7	2.12	1.30	[-2.34, 6.57]	1.62	1.000	0.53
M1 - P6	-3.84	1.29	[-8.23, 0.56]	-2.98	.211	-0.95
M1 - P8	-1.22	1.28	[-5.59, 3.16]	-0.95	1.000	-0.30
M1 - P9	3.61	1.28	[-0.78, 7.99]	2.81	.358	0.90
M1 - P10	-1.26	1.29	[-5.66, 3.15]	-0.98	1.000	-0.31
M1 - P11	1.06	1.29	[-3.34, 5.46]	0.83	1.000	0.26
M1 - P12	1.02	1.31	[-3.44, 5.49]	0.78	1.000	0.25
M2 - M3	2.30	1.29	[-2.12, 6.73]	1.78	1.000	0.57
M2 - M4	-6.59	1.30	[-11.04, -2.14]	-5.07	< .001	-1.64
M2 - M5	-1.55	1.29	[-5.96, 2.85]	-1.21	1.000	-0.39
M2 - M7	1.31	1.28	[-3.05, 5.68]	1.03	1.000	0.33
M2 - P6	-4.64	1.28	[-9.02, -0.26]	-3.62	.025	-1.15
M2 - P8	-2.02	1.28	[-6.39, 2.36]	-1.58	1.000	-0.50
M2 - P9	2.80	1.28	[-1.57, 7.18]	2.19	1.000	0.70
M2 - P10	-2.06	1.28	[-6.44, 2.32]	-1.61	1.000	-0.51
M2 - P11	0.26	1.30	[-4.20, 4.72]	0.20	1.000	0.06
M2 - P12	0.22	1.29	[-4.18, 4.62]	0.17	1.000	0.05
M3 - M4	-8.89	1.31	[-13.38, -4.41]	-6.78	< .001	-2.21
M3 - M5	-3.86	1.28	[-8.25, 0.53]	-3.01	.196	-0.96
M3 - M7	-0.99	1.30	[-5.44, 3.46]	-0.76	1.000	-0.25
M3 - P6	-6.94	1.30	[-11.39, -2.49]	-5.34	< .001	-1.72
M3 - P8	-4.32	1.30	[-8.75, 0.11]	-3.34	.067	-1.07
M3 - P9	0.50	1.28	[-3.88, 4.88]	0.39	1.000	0.12
M3 - P10	-4.36	1.28	[-8.74, 0.01]	-3.41	.052	-1.08
M3 - P11	-2.04	1.28	[-6.43, 2.34]	-1.59	1.000	-0.51
M3 - P12	-2.08	1.30	[-6.54, 2.37]	-1.60	1.000	-0.52
M4 - M5	5.04	1.32	[0.52, 9.55]	3.82	.012	1.25
M4 - M7	7.91	1.31	[3.43, 12.38]	6.04	< .001	1.96
M4 - P6	1.95	1.29	[-2.47, 6.38]	1.51	1.000	0.49
M4 - P8	4.57	1.28	[0.19, 8.96]	3.57	.029	1.14
M4 - P9	9.40	1.30	[4.95, 13.84]	7.23	< .001	2.33
M4 - P10	4.53	1.29	[0.11, 8.96]	3.50	.038	1.13
M4 - P11	6.85	1.31	[2.37, 11.33]	5.23	< .001	1.70
M4 - P12	6.81	1.31	[2.32, 11.30]	5.18	< .001	1.69
M5 - M7	2.87	1.30	[-1.59, 7.33]	2.20	1.000	0.71
M5 - P6	-3.08	1.30	[-7.53, 1.37]	-2.37	1.000	-0.77
M5 - P8	-0.46	1.30	[-4.91, 3.98]	-0.36	1.000	-0.11
M5 - P9	4.36	1.28	[-0.01, 8.73]	3.41	.052	1.08
M5 - P10	-0.50	1.29	[-4.91, 3.91]	-0.39	1.000	-0.13
M5 - P11	1.81	1.30	[-2.63, 6.26]	1.40	1.000	0.45
M5 - P12	1.77	1.28	[-2.61, 6.16]	1.38	1.000	0.44
M7 - P6	-5.95	1.28	[-10.34, -1.56]	-4.63	< .001	-1.48

Comparison	Adjusted mean difference	SE	Bonferroni-adjusted 95% CI	t	Adjusted *p*	Cohen's *d*
M7 - P8	-3.33	1.29	[-7.73, 1.07]	-2.59	.677	-0.83
M7 - P9	1.49	1.29	[-2.91, 5.89]	1.16	1.000	0.37
M7 - P10	-3.37	1.29	[-7.77, 1.02]	-2.62	.616	-0.84
M7 - P11	-1.05	1.31	[-5.55, 3.44]	-0.80	1.000	-0.26
M7 - P12	-1.09	1.30	[-5.54, 3.35]	-0.84	1.000	-0.27
P6 - P8	2.62	1.28	[-1.76, 7.00]	2.05	1.000	0.65
P6 - P9	7.44	1.28	[3.06, 11.83]	5.80	< .001	1.85
P6 - P10	2.58	1.29	[-1.83, 6.99]	2.00	1.000	0.64
P6 - P11	4.90	1.31	[0.41, 9.38]	3.73	.016	1.22
P6 - P12	4.86	1.30	[0.41, 9.30]	3.74	.016	1.21
P8 - P9	4.82	1.28	[0.44, 9.21]	3.76	.015	1.20
P8 - P10	-0.04	1.28	[-4.42, 4.34]	-0.03	1.000	-0.01
P8 - P11	2.28	1.30	[-2.15, 6.71]	1.76	1.000	0.57
P8 - P12	2.24	1.30	[-2.20, 6.68]	1.72	1.000	0.56
P9 - P10	-4.86	1.28	[-9.23, -0.49]	-3.81	.012	-1.21
P9 - P11	-2.55	1.29	[-6.97, 1.88]	-1.97	1.000	-0.63
P9 - P12	-2.59	1.29	[-6.98, 1.81]	-2.01	1.000	-0.64
P10 - P11	2.32	1.29	[-2.08, 6.72]	1.80	1.000	0.58
P10 - P12	2.28	1.30	[-2.16, 6.71]	1.76	1.000	0.57
P11 - P12	-0.04	1.32	[-4.57, 4.49]	-0.03	1.000	-0.01

Note. Pairwise comparisons were based on estimated marginal means obtained from the linear mixed-effects model adjusted for experimental day, trial order, session order, and session-baseline valence. The p values and confidence intervals were adjusted using the Bonferroni method across all 66 pairwise comparisons. Degrees of freedom were estimated using the Satterthwaite method and were 202.62 for all comparisons. Standardized effects were calculated by dividing each adjusted mean difference by the residual standard deviation of the model. A negative value indicates that the first condition had a lower estimated valence score than the second condition. M denotes movement; P denotes posture.

3 Supplementary Table S3
Pairwise comparisons of adjusted estimated marginal means for heart rate across conditions

Comparison	Adjusted mean difference	SE	df	Bonferroni-adjusted 95% CI	t	Adjusted *p*	Cohen's *d*
M1 - M2	16.10	2.68	202.67	[6.95, 25.26]	6.01	< .001	1.93
M1 - M3	17.21	2.68	202.67	[8.04, 26.37]	6.42	< .001	2.06
M1 - M4	-2.03	2.68	202.67	[-11.21, 7.15]	-0.76	1.000	-0.24
M1 - M5	29.45	2.69	202.67	[20.25, 38.65]	10.95	< .001	3.52
M1 - M7	30.69	2.71	202.67	[21.43, 39.94]	11.34	< .001	3.67
M1 - P6	30.54	2.70	208.20	[21.30, 39.79]	11.29	< .001	3.65
M1 - P8	52.54	2.69	208.24	[43.33, 61.75]	19.51	< .001	6.28
M1 - P9	35.75	2.70	208.22	[26.52, 44.97]	13.25	< .001	4.28
M1 - P10	44.20	2.71	208.18	[34.95, 53.46]	16.32	< .001	5.29
M1 - P11	28.45	2.71	208.19	[19.20, 37.70]	10.51	< .001	3.40
M1 - P12	48.43	2.74	208.05	[39.05, 57.81]	17.64	< .001	5.79
M2 - M3	1.10	2.69	202.67	[-8.09, 10.29]	0.41	1.000	0.13
M2 - M4	-18.14	2.70	202.67	[-27.37, -8.90]	-6.72	< .001	-2.17
M2 - M5	13.35	2.68	202.67	[4.19, 22.51]	4.98	< .001	1.60
M2 - M7	14.58	2.65	202.67	[5.51, 23.65]	5.50	< .001	1.74
M2 - P6	14.44	2.69	208.23	[5.23, 23.65]	5.36	< .001	1.73
M2 - P8	36.44	2.69	208.24	[27.23, 45.64]	13.53	< .001	4.36
M2 - P9	19.64	2.69	208.25	[10.45, 28.84]	7.30	< .001	2.35
M2 - P10	28.10	2.70	208.23	[18.89, 37.31]	10.43	< .001	3.36
M2 - P11	12.34	2.74	208.07	[2.98, 21.71]	4.51	< .001	1.48
M2 - P12	32.33	2.70	208.20	[23.08, 41.57]	11.96	< .001	3.87
M3 - M4	-19.24	2.72	202.67	[-28.55, -9.93]	-7.06	< .001	-2.30
M3 - M5	12.24	2.67	202.67	[3.13, 21.36]	4.59	< .001	1.46
M3 - M7	13.48	2.71	202.67	[4.23, 22.73]	4.98	< .001	1.61
M3 - P6	13.34	2.73	208.08	[3.99, 22.69]	4.88	< .001	1.60
M3 - P8	35.33	2.72	208.12	[26.02, 44.65]	12.97	< .001	4.23
M3 - P9	18.54	2.69	208.24	[9.34, 27.74]	6.89	< .001	2.22
M3 - P10	27.00	2.69	208.25	[17.80, 36.19]	10.04	< .001	3.23
M3 - P11	11.24	2.70	208.22	[2.02, 20.47]	4.17	.003	1.34
M3 - P12	31.23	2.74	208.07	[21.86, 40.59]	11.40	< .001	3.73
M4 - M5	31.48	2.74	202.67	[22.11, 40.86]	11.48	< .001	3.77
M4 - M7	32.72	2.72	202.67	[23.42, 42.01]	12.04	< .001	3.91
M4 - P6	32.58	2.72	208.13	[23.27, 41.88]	11.97	< .001	3.90
M4 - P8	54.57	2.69	208.23	[45.36, 63.78]	20.25	< .001	6.53
M4 - P9	37.78	2.73	208.10	[28.44, 47.12]	13.83	< .001	4.52
M4 - P10	46.24	2.72	208.13	[36.93, 55.54]	16.99	< .001	5.53
M4 - P11	30.48	2.75	208.02	[21.07, 39.89]	11.08	< .001	3.65
M4 - P12	50.46	2.76	207.99	[41.02, 59.91]	18.27	< .001	6.04
M5 - M7	1.24	2.71	202.67	[-8.03, 10.50]	0.46	1.000	0.15
M5 - P6	1.09	2.73	208.08	[-8.26, 10.44]	0.40	1.000	0.13
M5 - P8	23.09	2.73	208.09	[13.75, 32.43]	8.45	< .001	2.76
M5 - P9	6.30	2.69	208.25	[-2.90, 15.49]	2.34	1.000	0.75
M5 - P10	14.75	2.71	208.17	[5.49, 24.02]	5.44	< .001	1.76
M5 - P11	-1.00	2.73	208.08	[-10.35, 8.35]	-0.37	1.000	-0.12
M5 - P12	18.98	2.70	208.22	[9.76, 28.20]	7.04	< .001	2.27

Comparison	Adjusted mean difference	SE	df	Bonferroni-adjusted 95% CI	t	Adjusted *p*	Cohen's *d*
M7 - P6	-0.14	2.70	208.21	[-9.38, 9.09]	-0.05	1.000	-0.02
M7 - P8	21.85	2.70	208.20	[12.61, 31.10]	8.08	< .001	2.61
M7 - P9	5.06	2.71	208.18	[-4.19, 14.32]	1.87	1.000	0.61
M7 - P10	13.52	2.70	208.20	[4.28, 22.76]	5.00	< .001	1.62
M7 - P11	-2.24	2.76	207.99	[-11.68, 7.20]	-0.81	1.000	-0.27
M7 - P12	17.75	2.73	208.10	[8.41, 27.08]	6.50	< .001	2.12
P6 - P8	22.00	2.66	202.67	[12.91, 31.08]	8.28	< .001	2.63
P6 - P9	5.20	2.66	202.67	[-3.91, 14.31]	1.95	1.000	0.62
P6 - P10	13.66	2.68	202.67	[4.50, 22.82]	5.10	< .001	1.63
P6 - P11	-2.10	2.72	202.67	[-11.41, 7.22]	-0.77	1.000	-0.25
P6 - P12	17.89	2.70	202.67	[8.66, 27.12]	6.63	< .001	2.14
P8 - P9	-16.79	2.66	202.67	[-25.90, -7.68]	-6.30	< .001	-2.01
P8 - P10	-8.34	2.66	202.67	[-17.44, 0.77]	-3.13	.132	-1.00
P8 - P11	-24.09	2.69	202.67	[-33.30, -14.89]	-8.95	< .001	-2.88
P8 - P12	-4.11	2.70	202.67	[-13.33, 5.11]	-1.52	1.000	-0.49
P9 - P10	8.46	2.65	202.67	[-0.62, 17.53]	3.19	.110	1.01
P9 - P11	-7.30	2.69	202.67	[-16.48, 1.88]	-2.72	.471	-0.87
P9 - P12	12.68	2.67	202.67	[3.55, 21.82]	4.75	< .001	1.52
P10 - P11	-15.76	2.67	202.67	[-24.89, -6.62]	-5.90	< .001	-1.88
P10 - P12	4.23	2.69	202.67	[-4.98, 13.44]	1.57	1.000	0.51
P11 - P12	19.98	2.75	202.67	[10.58, 29.39]	7.27	< .001	2.39

Note. Pairwise comparisons were based on estimated marginal means obtained from the linear mixed-effects model adjusted for experimental day, trial order, session order, and session-baseline heart rate. The p values and confidence intervals were adjusted using the Bonferroni method across all 66 pairwise comparisons. Degrees of freedom were estimated using the Satterthwaite method. Standardized effects were calculated by dividing each adjusted mean difference by the residual standard deviation of the model. A negative value indicates that the first condition had a lower estimated heart rate than the second condition. M denotes movement; P denotes posture.
